# Visualization of bone formation in sheep’s middle ear by using fluorochrome sequential labelling (FSL)

**DOI:** 10.1038/s41598-024-57630-w

**Published:** 2024-03-25

**Authors:** Max Kemper, Anne Kluge, Michael Ney, Thomas Beleites, Ines Zeidler-Rentzsch, Christiane Keil, Thomas Zahnert, Marcus Neudert

**Affiliations:** 1 Department of Otorhinolaryngology Head and Neck Surgery, Faculty of Medicine (and University Hospital) Carl Gustav Carus, Technische Universität Dresden, Fetscherstrasse 74, 01307 Dresden, Germany; 2 Department of Audiology and Phoniatrics, Charité – University Medicine Berlin, Berlin, Germany; 3 Department of Orthodontics, Faculty of Medicine (and University Hospital) Carl Gustav Carus, Technische Universität Dresden, Fetscherstrasse 74, 01307 Dresden, Germany

**Keywords:** Mammalian middle ear, Bone formation, Osseoregenerative process, Stapes footplate, Fluorochrome sequence labelling, Anatomy, Bone imaging, Fluorescence imaging, Optical imaging

## Abstract

One factor for the lacking integration of the middle ear stapes footplate prosthesis or the missing healing of stapes footplate fractures could be the known osteogenic inactivity. In contrast, it was recently demonstrated that titanium prostheses with an applied collagen matrix and immobilised growth factors stimulate osteoblastic activation and differentiation on the stapes footplate. Regarding those findings, the aim of this study was to evaluate the potential of bone regeneration including bone remodeling in the middle ear. Ten one-year-old female merino sheep underwent a middle ear surgery without implantation of middle ear prostheses or any other component for activating bone formation. Post-operatively, four fluorochromes (tetracycline, alizarin complexion, calcein green and xylenol orange) were administered by subcutaneous injection at different time points after surgery (1 day: tetracycline, 7 days: alizarin, 14 days: calcein, 28 days: xylenol). After 12 weeks, the temporal bones including the lateral skull base were extracted and histologically analyzed. Fluorescence microscopy analysis of the entire stapes with the oval niche, but in particular stapes footplate and the *Crura stapedis* revealed evidence of new bone formation. Calcein was detected in all and xylenol in 60% of the animals. In contrast, tetracycline and alizarin could only be verified in two animals. The authors were able to demonstrate the osseoregenerative potential of the middle ear, in particular of the stapes footplate, using fluorescence sequence labelling.

## Introduction

The aim of reconstructive middle ear surgery is to achieve optimum hearing rehabilitation and long-term stable results. Both objectives are dependent on the stable and secure positioning of the total ossicular replacement prosthesis (TORP) in the center of the stapes footplate, sufficient prosthesis length to ensure tension between the ossicular chain remnants/ tympanic membrane and the footplate with sufficient resilience (pre-tension of the annular ligament). On the other hand, a sufficiently tight fit of the prosthesis is necessary to prevent dislocation. To date, the surrounding tissue, the production of a cartilage shoe or the Omega connector have been available to secure the position of the prosthesis^1–4^. These techniques can prevent horizontal dislocation, but the interposition of tissue reduces sound transmission and the risk of prosthesis elevation in the perpendicular direction is not eliminated. This results in the need for a compound prosthesis concept in two steps. In the first step, a footplate anchor with surface modifications would be implanted after rehabilitation of the ear and the Columella prosthesis would be inserted in a second surgical step after bony integration of the prosthesis anchor^5,6^. This could achieve both a long-term stable result and optimal sound transmission. For this a fixed implant on the stapes footplate, with active bone formation as the main condition, in addition to avoiding dislocation of the prosthesis is necessary. Many types of prostheses materials, as well as diverse ossicular chain reconstruction techniques, were tested in the middle ear^7^. To date, the problems of progressive hearing loss and prosthesis dislocation after otosurgery have not been resolved satisfactorily. One reason for the absent integration of the middle ear stapes footplate prostheses or the failed healing of stapes footplate fractures could be the osteogenic inactivity of this middle ear region. Development of the ossicular chain in mammals is prematurely completed in the fetal period^8^, and thus the osseous potential for new bone formation may be limited for osseointegration. It has been described that repair processes focusing on the ossicular chain do not exist in the postnatal period and previous fractures do not heal^9^. Does and Bottema provide evidence of fracture remnants after previous skull base fractures dating back two to twenty years. They described that these patients with posttraumatic damage to the ossicular chain have no bone formation within the bony defect. This phenomenon can be explained by the lack of growth potential in the postnatal period, and the completed footplate growth at the end of the fourth embryonic month^10–15^. But Sudhoff et al. reported for the first time about a bony integrated titanium TORP on the footplate, which was noticed during a revision tympanoplasty because the stapes footplate was accidentally extracted. In addition, the fixation of the ossicular chain by bone dust was discovered in mastoid revision surgeries^16,17^.

To examine any potential bone turnover, polychrome fluorochrome sequence labelling (FSL) has already been used in mammals. This procedure uses calcium-binding fluorescent dyes to investigate bone formation and remodeling, and eventually results in visualisation of osseointegration and general bone metabolism^18–21^. Previous studies demonstrated the use of fluorochrome labelling in the bone remodeling of the tibia, skull, mandible, and maxilla^22–24^. Most of them were animal experiments with mice or other mammals^25^. The studies show bone remodeling in critical and non-critical defect sizes and estimate the potential and behaviour of bone turnover. Bone regeneration could be detected using biomaterials in both critical and non-critical defect sizes on long and skull bones^26–28^. Dost et al. demonstrated that human osteoblast-like cells of the stapes proliferate in the presence of different biomaterials such as aluminium-ceramics, gold, and even titanium^29^. Only a few animal experiments were published about the integration of prostheses into the ossicular chain. Bone integration of Ceravital and hydroxyapatite on guinea pigs´ stapes was observed after three months^30^. In contrast, no osseous connection between polycell and Ceravital on the rabbits´ stapes footplate was found, and no bone integration was found in guinea pigs^31,32^. Generally, in the middle ear, the relevance and distribution patterns of the osseointegration are largely unknown. Neudert et al. demonstrated that titanium prostheses with applied collagen matrix and immobilized growth factors stimulate osteoblastic activation and differentiation on the stapes footplate^5,6^.

This study aims to identify and accurately evaluate the potential of osseous regeneration including newly formed bones as well as bone remodeling, and the total potential of osseointegration in the middle ear. Fluorochrome sequence labelling was used to detect bone turnover in the middle ear.

## Material and methods

### Animals

All methods were carried out in accordance with relevant guidelines and regulations. All animal testing was conducted according to the ARRIVE Guidelines. The Animal Care Committee of the State of Saxony approved the study (Landesdirektion Sachsen, Germany, Aktenzeichen: 24D-9168.11–1-2005–14).

Ten 1-year-old female Merino sheep with a mean weight of 52 kg were obtained from a local breeder (Theinert, Canitz, Germany) and kept in an appropriate manner. All animals had free access to water and standard commercial feed. One week before surgical intervention, the animals were transferred to the local animal experimentation center (MTZ Dresden, Germany) and were returned to the local breeder one-week post-surgery. For all experimental procedures, except fluorochrome administration, the animals were placed in the local animal experimentation center.

### Surgical procedure

The sheep were premedicated with 0,6 mg/kg body weight Midazolam s.c. (Parker-Davis, Berlin, Germany) and 10–20 mg/kg Thiopental i.v. (Parker-Davis, Berlin, Germany). Following premedication, the trachea was intubated with a cuffed 8.0-mm internal diameter endotracheal tube and mechanically ventilated with a mixture of oxygen and nitrous oxide (1:2). An admixture of 1,8 vol% isoflurane with a tidal volume of 6 ml/kg was administered. Muscle paralysis during mechanical ventilation was achieved by administering 0.6 mg/kg ruconiumbromid (N.V. Organon, Netherlands). Analgesia was performed during the intervention with 35 mg/kg metamizole i.v. and postoperatively with 0.005 mg/kg buprenorphine s.c. For infection prophylaxis, 15 mg/kg amoxicillin s.c. (Duphamox LA, Forte Dodge) was administered after the surgery.

During the bilateral middle ear surgery, the ossicular chain was prepared via the transmeatal approach. In both middle ears, the incudostapedial joint was separated and the incus removed. In this way, the entire stapes could be inspected to rule out possible pathologies. With the help of the microscope, pathologies such as a fracture, immobility of joint between stapes and incus, tympanosclerosis, bony malformations or otosclerosis were excluded. The stapes was inspected to identify possible pathologies. One middle ear was then closed without further manipulation, while the other was implanted with a prosthesis on the footplate. The present study refers exclusively to the results of the bony remodeling processes of the stapes without an implanted prosthesis. The basic ability to form new bone in the middle ear, especially in the ossicular chain or stapes, is to be investigated. The results of the osseointegration of middle ear prostheses have already been published by Neudert et al^6^.

### Experimental procedure

**Figure 1:**
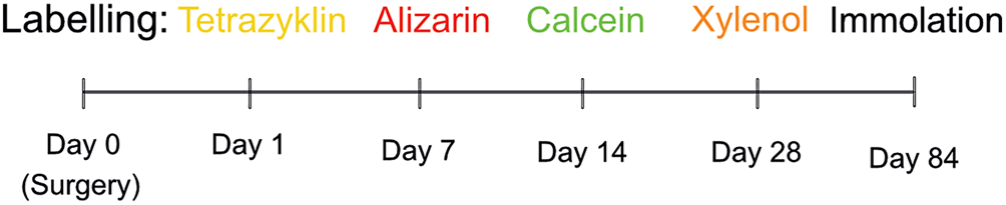
Time course of interventions and labelling procedure.

To study the bone formation and remodeling in various periods of bone growth, four calcium-binding fluorescent dyes [tetracycline (20 mg/kg BW), alizarin complexone (30 mg/kg BW), calcein green (20 mg/kg BW), and xylenol orange (90 mg/kg BW)] were administered at the below-mentioned postoperative intervals (Fig. 1) as described previously^33^. To demonstrate the incorporation of fluorochromes into the regenerated bone tissue, part of the tibial shaft was also in some sheep explanted and analyzed. The current literature and own previous experiments show that bone formation occurs mainly in the second and third week after an intervention or trauma. Tetracycline was used as a postoperative antibiotic on day 1 after surgery.

### Histology

At the end of the observation period (week 12), the animals were terminated by intravenous single injection (5 ml/50 kg) of Embutramide (200 mg/ml), tetracainhydrochloride (5 mg/ml), and mebezoniumiodide (50 mg/ml) (T61®, Intervet Co, Unterschleißheim, Germany). The temporal bones were extracted, including the lateral skull base as en bloc resection. In addition, part of the tibia was removed from three sheep to confirm the feasibility of staining the long bone. The specimens were fixed in 4% neutral buffered formaldehyde solution until further preparation. The fixed bone samples were reduced to a size of about 1.5 cm^3^. In order to recognize the stapes, in the next step, the stapes was marked by placing a destination cylinder on the oval niche. After conventional embedding in methylmethacrylate (Technovit 7200, Kulzer, Wehrheim, Germany), each sample was ground until the entire stapes appeared at the specimen’s surface using the cutting-grinding technique according to Donath and Breuer^34^.

Photomicrographs at different magnifications were obtained from the groundspecimens using a fluorescence microscope (BX 61, Olympus, Hamburg, Germany).

Next, the Masson–Goldner histological sections were stained, and light microscopic images were prepared by observing identical locations. Thereafter, the sample was processed sequentially 3–4 times as described above, starting with a new reduction of 100 μm, thereby taking 3–4 images per specimen showing the entire stapes.

## Results

### Detection of fluorochrome incorporation in the tibial shaft

**Figure 2:**
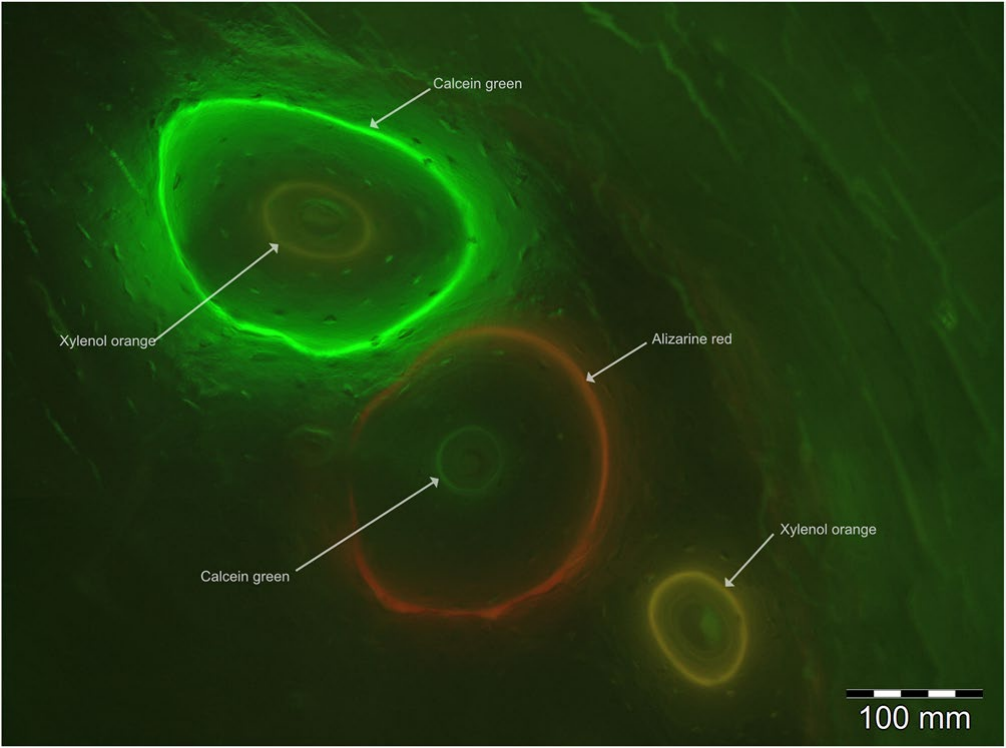
Fluorescence microscopic image of the tibial shaft. Yellow fluorescence: tetracycline, red fluorescence: alizarin, green fluorescence: calcein green, orange fluorescence: xylenol orange, HC: Haversian canal, magnification: × 200.

In this study, four fluorochromes were subcutaneously administered in specific time intervals (Fig. 1). Figure 2 shows different colored fluorescent osteons in the tibia of a sheep. In total, the tibia of three sheep was analyzed as a positive control for the fluorescent labelling. Two ostia clearly show that new bone formation from the inside to the outside has occurred (two differently coloured fluorescent rings). One of these osteons shows a large outer green (calcein, day 14) and a small inner orange (xylenol, day 28) fluorescent ring. The other osteon has an outer red (alizarin, day 7) and an internal green (calcein, day 14) fluorescent label. A third bone lamella is only labeled with the fluorescent dye xylenol (orange, day 28). The Haversian canal, which is responsible for nutrient supply and stimulus transmission during bone formation and regeneration, can be seen in the center of these bone lamellae (Fig. 2).

### Detection of new bone formation in sheep stapes

**Figure 3:**
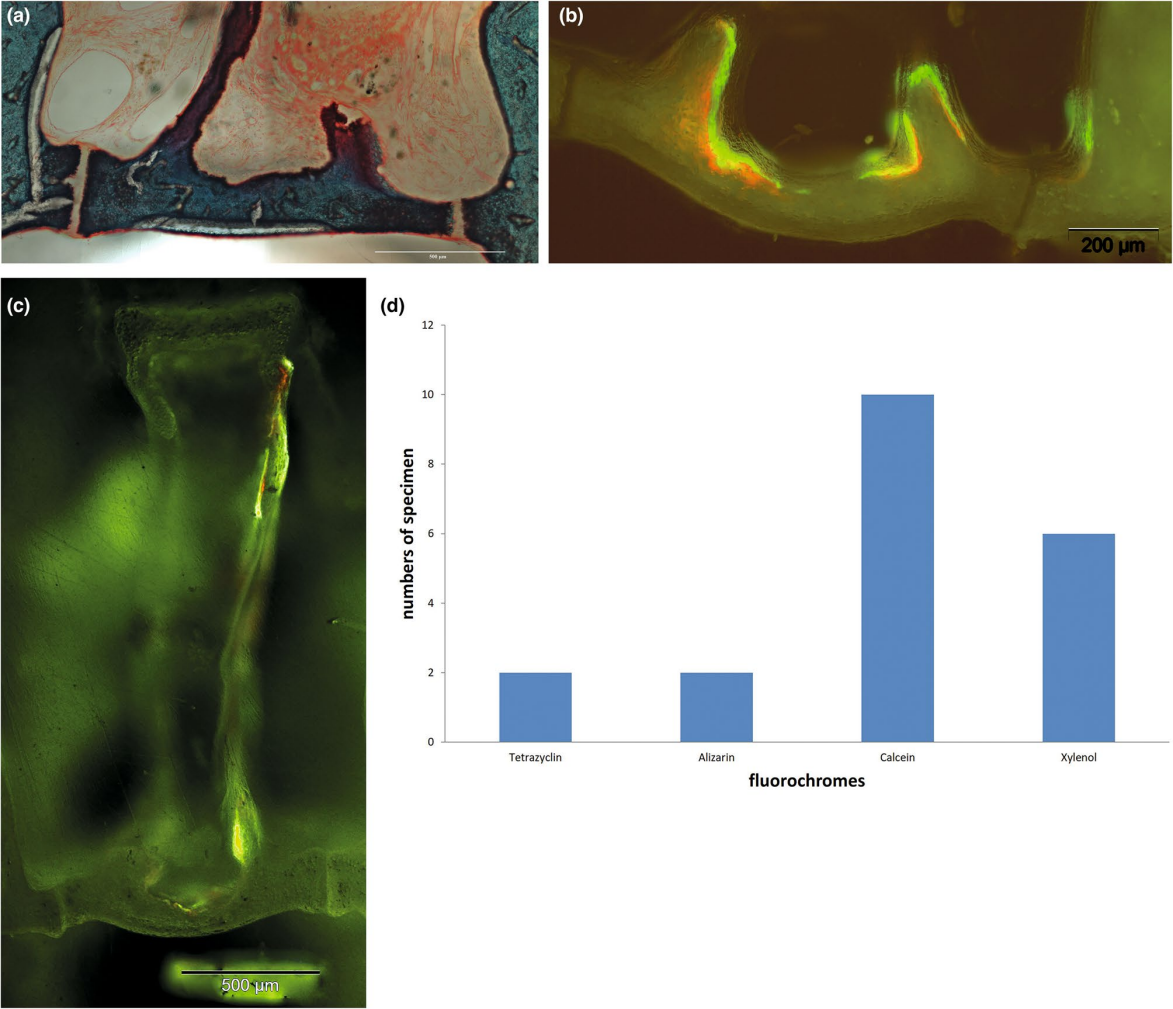
Bone formation in stapes footplate region. (**A**) Masson–Goldner staining, magnification: × 100, a cross-section of a stapes footplate region with a dissected stapes, the oval niche is completely filled with blood vessels, connective tissue, and collagen fibers (orange staining); (**B**) Fluorescence microscopic picture of stapes footplate, magnification: × 100, fluorochromes alizarin (red) and calcein (green); areas with bone regeneration (footplate and connection from the footplate to the legs), areas without bone regeneration (central footplate). (**C**) Fluorescence microscopic picture of *Crura stapedis*; magnification: × 40; fluorochromes alizarin (red) and calcein (green). (D) Quantification of the detection frequency of the applied fluorochromes. Graphs show absolute numbers of proofs.

Figure 3A shows a cross-section of a stapes footplate region with a dissected stapes. The bony footplate is clearly visible. The oval niche is completely filled with blood vessels, connective tissue, and collagen fibers.

Fluorescence microscopy analysis of the same region revealed evidence of new bone formation at the stapes footplate. Clearly visible are the fluorochromes alizarin (red, day 7) and calcein (green, day 14). The footplate shows no homogeneous distribution of fluorescence. Areas with bone regeneration and areas without bone regeneration can be observed (Fig. 3B).

In addition to the footplate, red and green fluorescence can be detected on the *Crura stapedis* (Fig. 3C).

The quantification of fluorochrome incorporation into the footplates revealed that calcein and xylenol are detectable in 10 and 6 animals, respectively. In contrast, tetracycline and alizarin could only be detected in 2 animals (Fig. 3D).

## Discussion

The main finding of the present study has shown that bone remodeling processes take place mainly 14 and 28 days after middle ear surgery on the stapes footplate, especially at its base in the angle between the footplate and the crura. To our knowledge, this is the first study addressing bone turnover, in particular bone formation and bone remodeling in the stapes footplate. In order to demonstrate a complete bone remodeling process, we opted to analyse fluorescence incorporation at week 12 after surgery. From studies on different animals and humans, it is known that bone regeneration takes place within 8–12 weeks^22,35,36^. Dumont et al. showed through polychromatic fluorescence the induction of bone healing after saw osteotomy compared to the normal fracture healing in the sheep tibia model^22^. For this reason, the tibia was used as a positive control for the fluorescent labelling in the study presented here. In the tibial shaft, the temporal course of bone regeneration was well recognized. Not all dyes are detectable in each osteon. This circumstance is due to the different states of osteon activity at the time of dye administration. This provides evidence that bone remodeling can be detected by FSL in the tibia and should therefore be detectable in other bones as well.

For several years, there has been a discussion about osseous regenerative procedures in the middle ear. So far, bone regeneration in the middle ear has been ruled out due to the fact that the development of the ossicular chain in mammals is prematurely completed in the fetal period^8^. Does and Bottema (1965) described absent bone healing after a fracture of the ossicular chain^9^. In contrast to these findings, it was recently shown that titanium prostheses with a modified surface (collagen matrix and immobilized BMP4) can be osseointegrated on the stapes footplate^6^. A fixed prosthesis on the footplate of the stapes is a prerequisite for satisfactory hearing results after ossiculoplasty^7,37–39^. In the area of the middle ear, especially on the stapes footplate, the patch or clamp technique cannot be used. Therefore, the secure fixation of prostheses on the footplate can be achieved only by bundling the connective tissue or cartilage guidance^1,6^. These techniques can prevent horizontal dislocation, but the interposition of tissue reduces sound transmission and the risk of prosthesis elevation in the perpendicular direction is not eliminated. This could be avoided by the bi-modular prosthesis concept (TORP + footplate anchor)^3^. For an optimal connection of a titanium footplate anchor on the stapes footplate or ossicular remnants, a permanent fixation on the stapes footplate is indispensable. This means that prostheses osseointegrate on the footplate. However, osseointegration in turn requires bone remodeling processes. The method mentioned allows bone turnover, especially bone remodeling and bone formation, which can be demonstrated on the stapes footplate and the crura without any further influencing factors. In addition, bone formations can also be detected in other areas of the middle ear^40^. Ossification lines also appeared in the region of the bony labyrinth capsule using this method. Thus, the activity of the bone metabolism in the middle ear and on the stapes footplate were shown. Whether the behaviour of the bone metabolism can be accurately transferred from the sheep´s middle ear to the human middle ear remains unclear. However, the sheep is also a mammal and has been used in numerous animal models to study bone metabolism and in the related research of osseous metabolic diseases^41,42^. Furthermore, the sheep model has been widely used to study localized fracture healing and integration of prosthetic devices in the skeleton, but not yet in the middle ear^43,44^. In the fluorescence microscopic view of our study, ossification lines appeared with enrichments of calcein green and xylenol orange. This implies that active bone turnover takes place mostly after two and three weeks because the fluorochromes are only bound to the calcium during active new bone formation. This fact is in line with current literature showing that bone formation occurs mainly in the second and third week after an intervention or trauma^33,45^. Thus, the FSL is a qualified method to represent bone formation and remodeling also in the middle ear. These results are of fundamental importance because this study shows that the middle ear and stapes are not static systems. In the meantime, these systems are constantly changing, especially when their integrity is disturbed in the context of trauma or surgery. But the lack of integration of the middle ear prosthesis may be because the trauma on the stapes footplate is too low for sufficient activation of bone turnover. This results in wound healing after such trauma with insufficient osseointegration of the prosthesis and thus no secure fixation of the prosthesis on the stapes footplate (comparison to other effects in traumatology). Therefore, other factors influencing osseointegration must be identified. From an embryological point of view, the stapes and thus the stapes footplate can be compared with the mandibular condyle since these structures develop from the second pharynx pharyngeal arch. In addition, it is known that the mandibular condyle has developed due to a secondary growth centre and endochondral growth. Furthermore, it could be shown that the inserting muscles cause a growth stimulus, which leads to the further development of the condyles. The structure of the condyle mandibulae resembles the stapes footplate because in both structures bone is covered by cartilage. For more accurate histological analysis, the stapes footplate consists of one-third bone and the remainder of a cartilaginous layer, creating a multi-layered structure^6,46^. But the layers of the stapes footplate are much thinner compared to the mandibular condyle since histological investigations show that the footplate is only 100 to 150 µm thick overall^6,47^. Apart from the tendon of the stapedius muscle, no other muscles insert on the stapes or notably the stapes footplate. Although it could be shown that even in these fine structures continuous bone turnover takes place, this is not always sufficient, for example, for the osseointegration of a prosthesis. This often requires a sufficiently large stimulus in the form of trauma.

In conclusion, in the present study using a sheep model it was demonstrated for the first time that bone turnover processes and new bone formation takes place in a non-irritated middle ear and especially at the stapes footplate by using fluorochromes sequence labelling. Because of the staining with four different fluorochromes, the greatest activity of the osteoblasts and thus the formation of new bone could be proven two to three weeks after application. Therefore, the thesis that the stapes footplate is a bioinert area can be refuted. Because of this, the bone in the middle ear on the stapes footplate seems to renew itself at regular intervals. However, fracture healing and osseointegration of prostheses require an additional sufficiently large activating stimulus^6^. Moreover, it is not yet known to what extent bone remodeling and thus osseointegration in the middle ear are influenced by age, gender, hormone status, medication, and various diseases, including bone metabolism.

### Limitation

In this study, active bone metabolism was visualized for the first time using fluorochrome sequence labelling, which enables the processes to be resolved over time. Due to the small size of the footplate and the method chosen to visualize bone metabolism, the preparation had to be prepared using the modified thin section technique according to Donath. Here, the tissue block to be analyzed is gradually ground down so that only reflected light microscopy could be performed. For a more detailed histological visualization of the (newly formed) bone, transmitted light microscopy using sectional preparations would be necessary, which could not be realized in this study design. The limitation of the study lies on the one hand in the small number of stapes specimens analyzed. As this study was carried out as part of a larger animal study, the number was considered a sufficiently large reference group. An increase in the number of specimens was not considered appropriate due to the consideration of the statistical significance and the ethical justifiability of killing additional animals. On the other hand, the method of fluorochrome fluorescence marking in conjunction with the histological processing method of the modified thin section technique according to Donath was chosen for this series of experiments. This method enabled a good visualization of the fluorochromes in the stapes, but meant that a histological evaluation could be carried out with the less precise reflected light microscopy. Transmitted light microscopy on sectional preparations, which is better for the histological imaging of bone and its new formation, was therefore not possible. Whether the bone metabolism processes observed on the basis of fluorochromes can be attributed exclusively to newly formed bone or to bone remodeling processes cannot ultimately be definitively proven. However, it can be established that in all 10 ears treated in the manner described, fluorochromes can be visualized with the corresponding progression over time. Therefore, an increased bone metabolism and thus new bone formation must be assumed. There is no other explanation for the ingrown prostheses described in the literature.

## Data Availability

Data sets generated and analyzed as part of the current study are not publicly available due to the size of the data and the associated lack of practicability, but are available upon reasonable request from the corresponding author.
